# Newborn Critical Congenital Heart Disease Screening Using Pulse Oximetry in a Global Context: Progress, Disparities, and the Importance of Early Detection

**DOI:** 10.3390/ijns12020031

**Published:** 2026-05-05

**Authors:** Lisa A. Hom, Gerard R. Martin

**Affiliations:** 1 Children’s National Heart & Lung Center, Children’s National Hospital, Washington, DC 20010, USA; gmartin@childrensnational.org; 2 The George Washington University School of Medicine and Health Sciences, Washington, DC 20052, USA

## 1. Introduction/Background

Congenital heart disease (CHD) remains the number one cause of mortality due to congenital defects in children under the age of one. Many critical forms present with hypoxemia and cardiovascular collapse in the early neonatal period, for which delayed diagnosis is a major contributor to mortality and morbidity [1,2]. Globally, CHD occurs in approximately 8–10 per 1000 live births, with roughly a quarter of those being critical forms requiring intervention during infancy [1,3,4]. While advances in our ability to diagnose and treat these infants have led to substantial improvements in survival in high-income countries, CCHD remains near the top of the list as a leading cause of death due to congenital anomalies globally [1].

Global mortality attributable to CCHD is marked by stark regional disparity. In their analysis of the Global Burden of Disease Study, Zimmerman et al. demonstrated that although mortality rates declined in high-income regions over the recent decades, the absolute burden of deaths remains the highest in low- to middle-income countries, where access to CCHD screening and specialized cardiac care is severely limited [1]. The greatest mortality burden occurs during infancy, which underscores the crucial role of early identification strategies like pulse oximetry screening (POS), capable of detecting CCHD prior to clinical deterioration.

Newborn CCHD screening using pulse oximetry emerged as a cost effective approach to addressing a diagnostic gap [5]. Pulse oximetry screening identifies at-risk infants with hypoxemia that were not identified as having CCHD through prenatal ultrasound or on routine physical exam following birth. It improves outcomes through earlier detection, thus reducing late presentation including circulatory collapse, and decreases infant mortality associated with CCHD, particularly at the population level [6]. These findings supported the widespread adoption and implementation of POS in many high-income countries and prompted further investigation and interest in implementation across diverse global healthcare systems [7,8].

## 2. Global Implementation: Progress and Persistent Disparities

**Figure 1:**
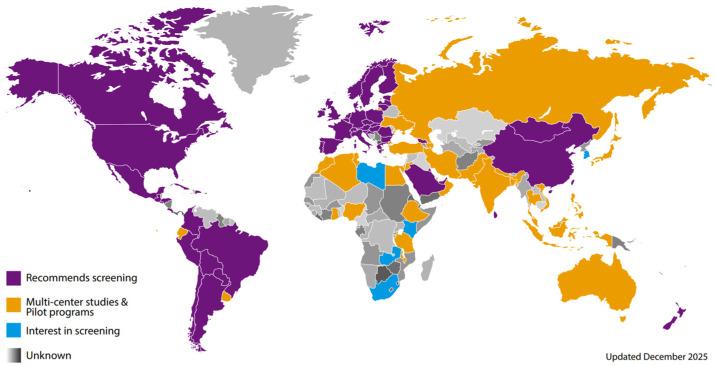
Global Critical Congenital Heart Disease Screening Map, last updated December 2025. This global implementation map was created based on published studies [2,9,13,14,15,16,17,18,19,20,21,22,23,24,25,26] and annual updates from the following international CCHD screening experts: Dr. Andrew Ewer, Dr. Anne Granelli, Dr. Augusto Sola, Dr. Gerard Martin, Lisa Hom, and Annamarie Saarinen.

The global expansion of CCHD screening has increased over the past two decades, though implementation remains regionally disparate. Using input from international CCHD screening experts and updated with published reports, countries were classified to reflect current CCHD screening implementation status or whether a national or regional recommendation by a professional society or governmental recommendation or mandate to screen is in place [Figure 1: Global Implementation Map]. In their global update, Abbas and Ewer describe substantial progress in the adoption of POS across multiple regions, along with persistent variability in program scope, sustainability, and integration into existing newborn screening and maternal health systems [7]. The difference described closely parallels global patterns of CCHD mortality, reinforcing the relationship between delayed diagnosis, the availability of health resources including the infrastructure to intervene once a diagnosis is made, and poor outcomes [9,10,11,12].

In North America and Europe, POS is very much a part of routine newborn care and in many countries is mandated or recommended at the regional, national, or state level [13,14,27]. In the United States, universal implementation has been associated with measurable reductions in early infant deaths attributable to CCHD [6]. Similar outcomes have been reported from Europe, where POS has reduced late diagnosis even in countries with high rates of prenatal detection [7,15,16,28].

Across Latin America and parts of the Middle East and North Africa, implementation has progressed through regional collaboration, professional society leadership (also true in the US and Europe), and center-based initiatives [17,18,19]. While national coverage remains limited in many countries, these efforts have demonstrated both feasibility and clinical value as well as identified challenges and solutions for middle-resource settings [18,20,21]. In contrast, screening remains limited to regional pilot and feasibility studies in much of sub-Saharan Africa and Asia—regions that bear a disproportionate share of global CCHD mortality [1,22,23]. Barriers in this region include early postnatal discharge, limited access to confirmatory echocardiography and pediatric cardiologists, lack of availability of surgical and interventional capacity—including cardiac intensive care post-operative recovery units, material and financial resources, other healthcare workforce constraints—and fragmented referral systems [8,23].

While CCHD screening in the United States and other western health systems is largely performed by nurses, midwives, technologists, or other trained hospital staff, reports from Middle East and North Africa (MENA) settings indicate that screening can be led and performed by physicians, including junior doctors or residents, reflecting local neonatal care structures and emphasizing the importance of context-specific implementation [16,24,29,30].

## 3. Prenatal Detection, Capacity, and Regional Outcomes

Prenatal detection of CCHD through fetal ultrasound or echocardiography improves perinatal planning, facilitates delivery at appropriate centers, and reduces early morbidity [31]. However, prenatal detection rates vary widely across regions and even within cities, reflecting disparities in access to prenatal care, trained providers, and advanced imaging modalities [7,32]. In many low-resource settings, the majority of infants with CCHD are born without a diagnosis, contributing to the high early mortality rates observed in these regions [9,23,25]. In regions where prenatal detection rates are low, the relative contribution of postnatal screening may be the greatest [33]. A significant proportion of infants with CCHD continue to be diagnosed only after birth, highlighting the complementary role of newborn pulse oximetry screening in improving early detection worldwide. As CCHD screening programs continue to expand globally, the relative contribution of postnatal screening may be the greatest in regions where prenatal care or prenatal ultrasound may not be systematically or uniformly available [26,33,34]. Conversely, a few centers with high prenatal detection rates and very few missed cases of CCHD prior to the implementation of CCHD screening report limited or no significant impact on the timing of diagnosis or mortality [35,36]. At the population level, mandatory screening is associated with a reduction in early CCHD deaths, although left outflow obstructive defects such as CoA and IAA continue to be challenging to detect [6,37].

Even in regions with high rates of prenatal detection, postnatal screening remains essential, as a substantial proportion of critical lesions continue to be missed antenatally [9,12,38]. The ability to intervene following diagnosis varies markedly by region. High-income countries with well-established systems for pediatric cardiac surgery and catheter-based interventions can provide excellent outcomes once CCHD is detected, whereas, in many low- and middle-income countries, specialized cardiac care remains very limited, centralized, or unavailable [8,22,25]. These disparities directly impact outcomes and highlight the importance of early diagnosis, even in places where intervention options are severely constrained [8,22].

While disparities in pediatric cardiac care infrastructure raise important considerations in settings where surgical or cardiac catheter-based interventions are limited, early identification of CCHD can still help families and clinical teams facilitate appropriate stabilization and referral pathways and engage in informed decision-making. Additionally, CCHD screening helps identify non-critical congenital heart conditions and other serious neonatal non-cardiac conditions such as neonatal sepsis, pneumonia, and other respiratory disorders that present with hypoxemia. Early detection of these conditions also enables timely diagnosis and treatment, contributing to further reductions in neonatal morbidity and mortality [9,13].

## 4. Health Equity and the Broader Benefits of CCHD Screening

Although the primary objective of POS is the early detection of CCHD, its benefits extend beyond cardiac disease. The broader impact of POS is in its ability to allow for the detection of other serious conditions causing hypoxemia. A significant proportion of infants who screen positive or fail their screen are ultimately diagnosed with conditions including neonatal sepsis, persistent pulmonary hypertension, and other respiratory and infectious disorders [27,34]. Early identification of these conditions is particularly important in regions with high neonatal mortality, where timely evaluation may be lifesaving and treatment options, such as antibiotics and fluids, may be more readily available than specialized pediatric cardiac interventions such as surgery [25,38,39].

In settings where neonatal death may otherwise occur without explanation, screening can help lead to diagnostic clarity for both clinical teams and families. Identifying an underlying cardiac or non-cardiac cause of symptoms or death can offer families understanding and closure, inform reproductive counseling, and support accurate public health surveillance and support systems [6,40,41]. In this context, CCHD screening provides not only an opportunity for the early identification of CCHD but also an equity-oriented intervention aligned with important global initiatives to reduce preventable neonatal deaths [7,9,38].

## 5. Conclusions

The implementation of pulse oximetry screening was named one of the 30 highest-impact innovations with the capacity to save lives as part of the Commitments to Support the World Health Organizations (WHO) “Every Woman Every Child Action Plan.” The estimated impact by 2030 is 772,000 child lives saved, including not just preventable CCHD deaths but a 6% reduction in deaths due to pneumonia [42,43].
